# Genome Sequence of Bacteriophage Myram Isola­­­ted With Host *Microbacterium foliorum*

**DOI:** 10.17912/micropub.biology.002004

**Published:** 2026-09-17

**Authors:** Solomon DeMello, Peyton Guay, Brooke Souder, Zach Stillman, Bekhruz Avganov, Jae Howell, Kit Williams, Emily Savage

**Affiliations:** 1 Biology, Southern Maine Community College, South Portland, ME USA

## Abstract

Bacteriophage Myram was isolated from sandy soil in South Portland, ME on *Microbacterium foliorum* NRRL B-24224. It has a siphovirus morphology and a 17,362-bp genome encoding 25 putative genes. Based on shared gene content, Myram is assigned to actinobacteriophage cluster EE.

**Figure 1. Plaque morphology and TEM of bacteriophage Myram:** (A) Plaques formed by phage Myram on *M. foliorum* NRRL B-24224 are an average of 2-4-millimeter (n=5) in diameter. (B) Virion of phage Myram by transmission electron microscopy (Hitachi HT7800, 120kV, accelerating voltage 100kV) using negative stain (1% uranyl acetate). Myram displays siphovirus morphology with a 40nm capsid and a tail length of 110nm (n=3) .

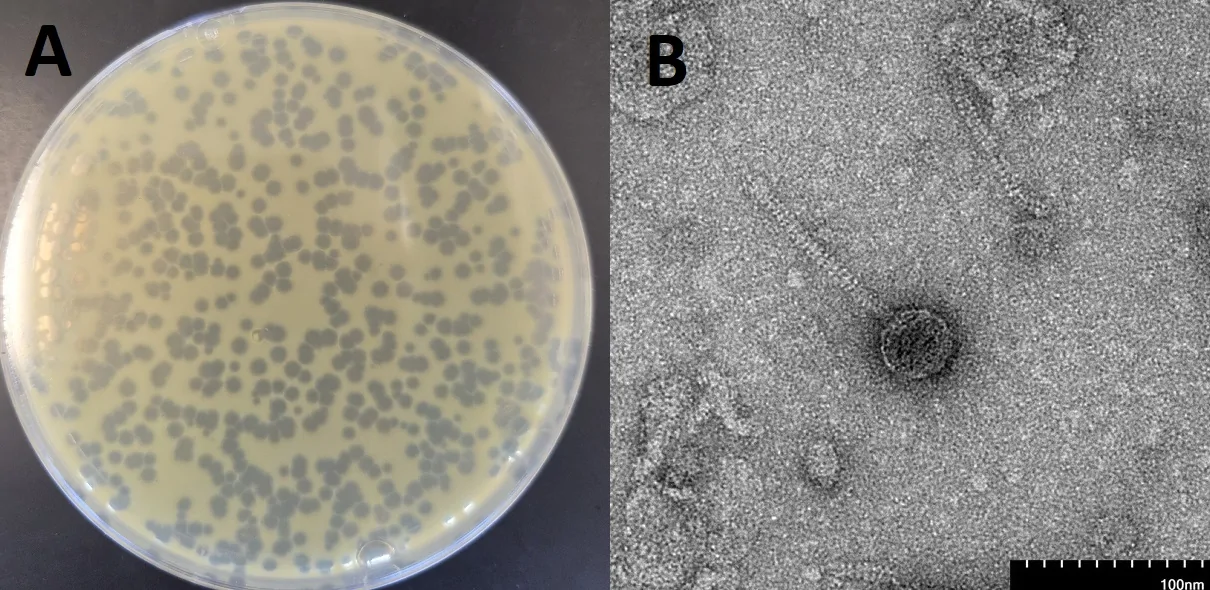



## Description

Actinobacteriophage are being developed for use in the treatment of multidrug-resistant bacterial infections (Hatfull 2020; Hibstu 2022). *Microbacterium foliorum* is an actinobacteria that is readily cultured, making it an ideal bacterium for the isolation and characterization of actinobacteriophages that can advance our understanding of actinobacteriophage biology and diversity and their application in medicine (Hatfull 2020; Russel et al., 2019).

Myram was isolated from sandy soil at the top of a cliff overlooking Willard Beach in South Portland, Maine (Global Positioning System 43.645555 N, 70.227498 E) using standard procedures (Zorawik et al., 2024). Five grams of soil were suspended in PYCa (peptone, yeast extract, and calcium chloride) liquid media and the suspension inoculated with *Microbacterium foliorum* NRRL B-24224, and incubated for 4 days at 30℃. The resulting culture was then filtered (0.22 µm pore size) and filtrate was spotted onto PYCa top agar with *M. foliorum* . Phage Myram formed clear plaques approximately 2-4mm (n=5) after 48 h at 30°C. Negative strain transmission electron microscopy revealed siphovirus morphology, with a capsid size of 40nm and a tail length of 110nm (n=3) (Figure 1).

Phage DNA was extracted from a lysate using the Promega Wizard DNA clean-up kit. The sequencing library was prepared using the NEB Ultra II Library Kit, then sequenced on the Illumina NextSeq 1000 sequencer with v3 reagents. This yielded 166M 100-base single-end reads, providing 9,347-fold coverage. Raw reads were trimmed and filtered by cutadapt 4.7 using the -nextseq-trim 30 option (Martin 2011), and skewer 0.2.2 using options -q 20 -Q 30 -n -l 50 (Jiang et al., 2014 ) and genomic termini and completeness was evaluated with Consed V29 (Gordon and Green, 2013) using default parameters. The complete Myram genome length is 17,362-bp with a 68.5% GC content and 3' single-stranded ends (5' CCCGCCCCA).

Myram's genome sequence was automatically annotated in DNA Master v5.23.6, build 2705 (Pope and Jacobs-Sera, 2017). GeneMark v2.5p (Besemer and Borodovsky, 2005) and Glimmer v3.02b (Delcher et al., 2007) were used to assess coding potential and identify protein-coding genes. BLASTp v2.16.0 using the Actinobacteriophage and the NCBI non-redundant protein sequences [nr] databases (Altschul et al., 1990), HHPred v2.08 using the PDB_mmCIF70, SCOPe70, Pfam-A, NCBI_Conserved_Domains [CD] databases (Söding et al., 2005), Phamerator v597, Actino_Draft(Cresawn et al., 2011) using the Actinodraft database, and Starterator v3.02(Pacey, 2016) were used to refine start selection and ascertain predicted gene functions. Aragorn v1.2.41.c. (Laslett and Canback, 2004) and tRNAscanSE v2.0.12 (Lowe and Eddy, 1997) were utilized to detect possible tRNAs. DeepTMHMM v1.2.33.c. (Hallgren et al., 2022) and SOSUI v1.11 (Hirokawa et al., 1998) were used for transmembrane domain detection. All software was operated on default settings. This analysis identified 25 putative protein-coding genes, of which 18 could be assigned a putative function.

Myram was assigned to cluster EE, based on gene content similarity (GCS) of at least 35% to phages in the same cluster (Pope et al.,2017; Russell and Hatfull, 2017). As with previously characterized cluster EE phages, Myram encodes structural proteins in the left third of the genome, a putative lysis cassette on the right arm containing an endolysin and two transmembrane proteins, and a predicted programmed translational frame shift encoding the tail assembly chaperones. All genes are transcribed unidirectionally, with the exception of three consecutive genes at the right end of the genome that encode for two DNA-binding proteins and an Lsr2-like DNA bridging protein. There are no identifiable integrase or immunity repressor functions suggesting that Myram is unlikely to establish lysogeny.

**Nucleotide sequence accession numbers**

The Myram sequence and annotation are available at GenBank with Accession No. PV876957 and Sequence Read Archive (SRA) No. SRX29714290 .
